# Large Unruptured Perineal Epidermoid Cyst: A Rare Case

**DOI:** 10.7759/cureus.30086

**Published:** 2022-10-09

**Authors:** SaiNidhi G Reddy, Pratap Parihar, Vadlamudi Nagendra, Neha Shetty, Bhagyasri Nunna

**Affiliations:** 1 Radiodiagnosis, Jawaharlal Nehru Medical College, Datta Meghe Institute of Medical Sciences, Wardha, IND

**Keywords:** keratin, surgery, mri, epidermoid cyst, perineal cyst

## Abstract

Perineal epidermoid lesions are uncommon, with only a few research papers accessible on the subject. Because these lesions are varied in origin and can range from benign to malignant, it can be difficult to tell them apart. A wide variety of lesions are evaluated in the differential diagnosis of perineal cystic lesions, however perineal epidermoid cyst is uncommon. An epidermoid cyst is a benign ectodermal congenital abnormality. Epidermoid cysts can be found all over the body, however, they are uncommon in the perineal area.

We discuss a case of a perineal epidermoid cyst in an adult female presenting to the general surgery department with painful perineal swelling. The patient was advised for an ultrasound (US) and MRI for further evaluation and the findings are reported.

## Introduction

EC(s) are benign tumors that usually affect the scalp, face, neck, back, and chest. It is usually asymptomatic when small in size and evolving, but these can become symptomatic as a result of secondary infection or when it grows to a size that displaces or causes a mass effect on the surrounding anatomical tissues [1]. Epidermal or epidermoid cysts form when epidermal cells proliferate in a confined dermal space. Misplaced ectodermal tissues during embryogenesis, obstruction of the pilosebaceous unit, or traumatic or surgical epithelial element implantation are all possible causes of epidermal cysts [2-4]. The anatomically variable and non-specific radiologic characteristics and symptoms of perineal cystic lesions make diagnosis challenging.

CT and MRI enable inspection of the entire intestinal wall layer as well as the perirectal tissue, making it easier to describe these abnormalities further. MRI has a strong radiologic diagnostic value since it can accurately show the perineal architecture. As a result, MRI has been largely employed to investigate perineal cystic lesions [5].

## Case presentation

A 45-year-old female patient presented to consult the department of general surgery with complaints of pain in the perianal region for three years. The symptoms had acutely worsened in the past week. There was no history of trauma to the patient. On local examination, there was a mild subcutaneous bulge in the natal cleft region. There was no evidence of a local rise in temperature or redness on inspection. The patient was advised of high-frequency soft tissue ultrasonography of the perianal area.

On ultrasonography of the perianal area, there was a well-defined cystic lesion with internal echoes within the subcutaneous plane. The patient was further advised to MRI pelvis in view of the complete evaluation of the lesion and to understand its extension.

Subsequently performed MRI showed a well-defined thin-walled, peripherally enhancing signal intensity lesion that was hyperintense on T2WI/STIR and isointense on T1WI measuring approximately 9.0 x 4.0 x 3.4 cm (AP x TRANS x COR) (Figure 2-6). The lesion was displacing the lower segments of the coccyx superiorly and abutting the posterior wall of the anus anteriorly. Posteriorly the lesion was extending up to the subcutaneous plane. The lesion showed no signs of local infiltration and the perilesional fascial planes are well visualized.

**Figure 1:**
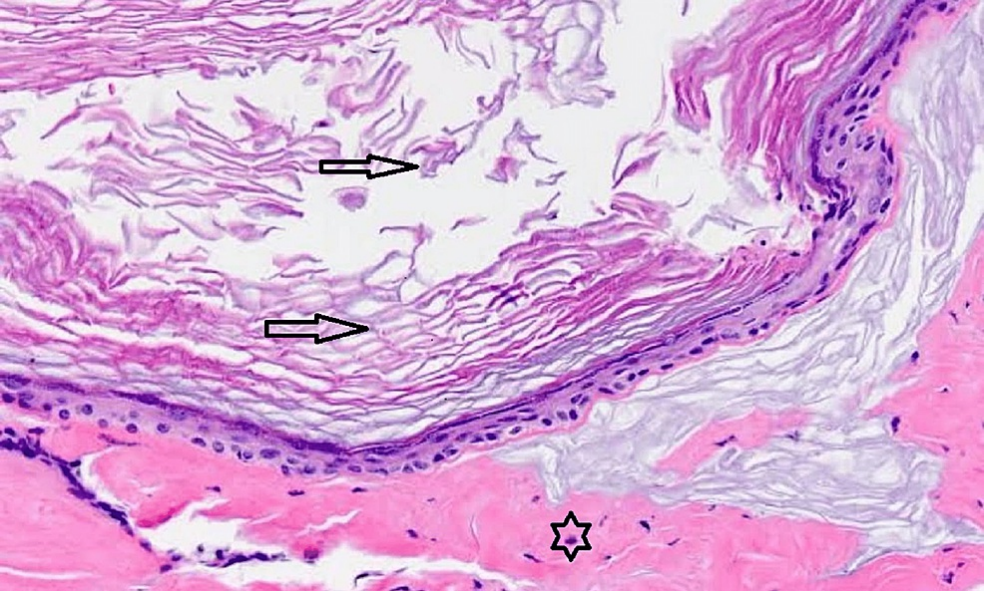
Histopathological findings show loose keratin flakes (black arrow) and epidermis (asterisk).

The patient was later advised for FNAC (fine needle aspiration cytology) which showed components of keratin (Figure 1). The patient was further posted for surgery and the post-operative sample was sent for evaluation confirming the epidermoid cyst.

**Figure 2:**
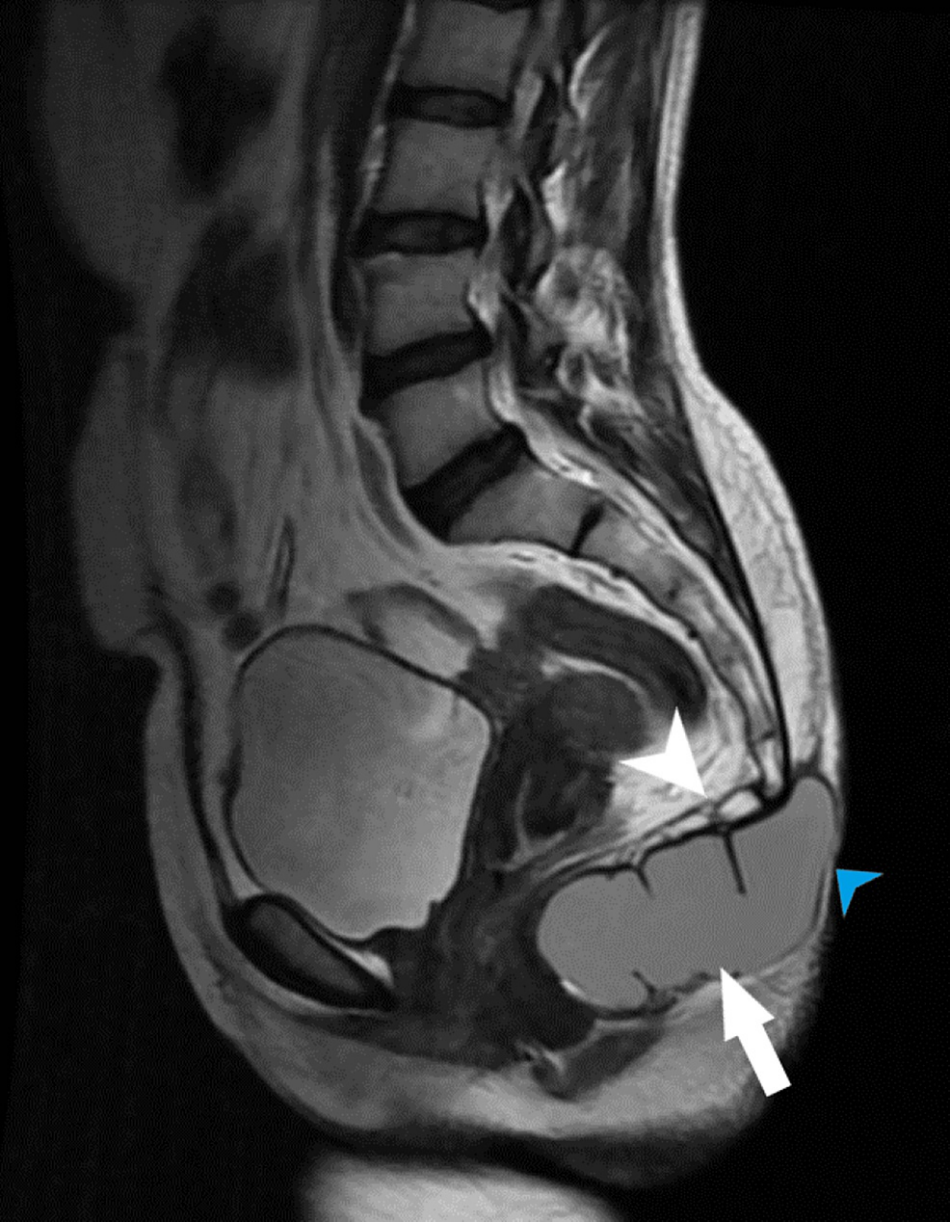
Sagittal T2WI image showing well-defined hyperintense lesion in the perineal region (white arrow), displacing the coccyx superiorly (white arrowhead). Posteriorly the lesion was extending up to the subcutaneous plane (blue arrowhead). T2WI: T2 weighted image https://assets.cureus.com/uploads/figure/file/450555/lightbox_97f08ff0313b11eda86031f47f95b255-E1.png

**Figure 3:**
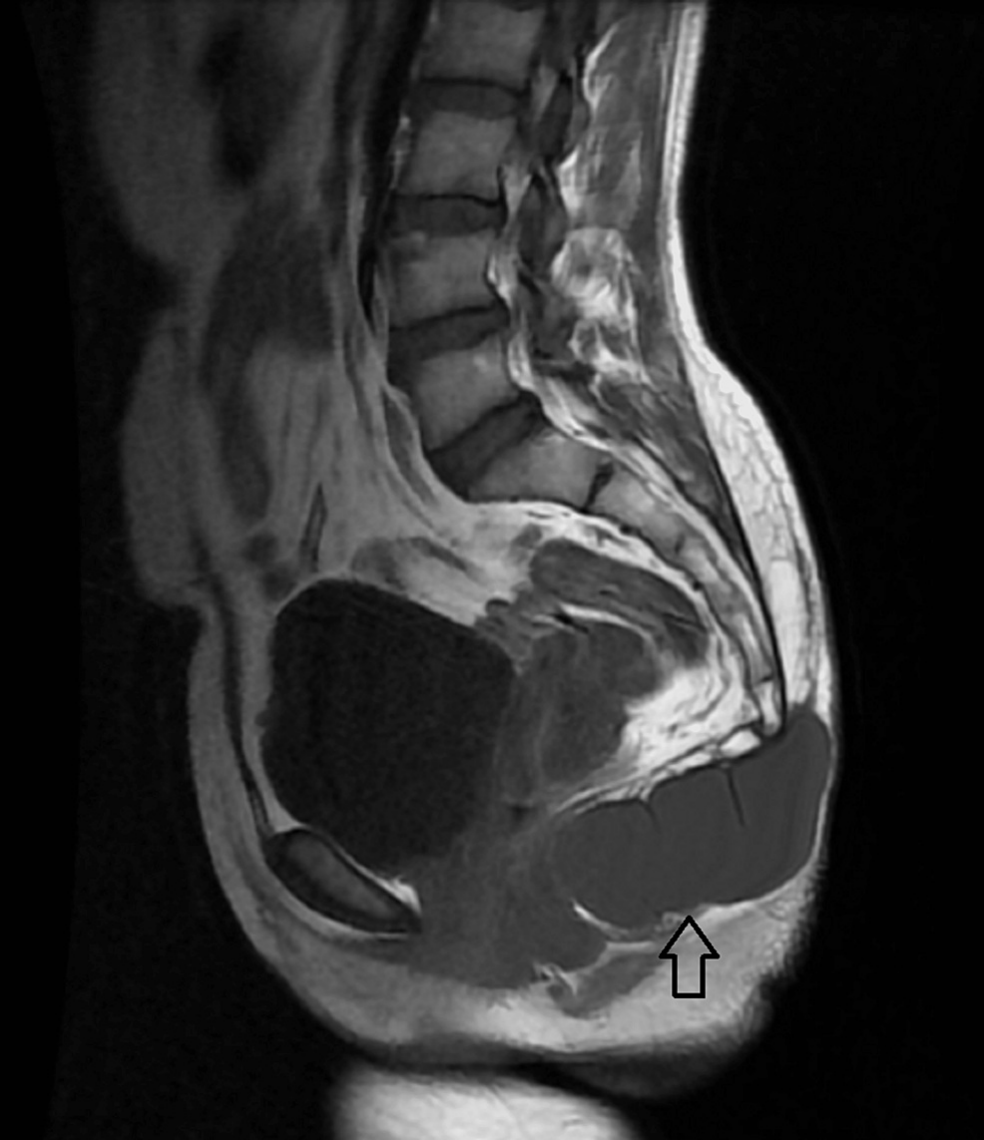
Sagittal T1WI MRI image showing well-defined isointense lesion in the perineal region (black arrow). T1WI: T1 weighted image https://assets.cureus.com/uploads/figure/file/450599/lightbox_8a119400313c11ed9d925f316fd743c0-E2.png

**Figure 4:**
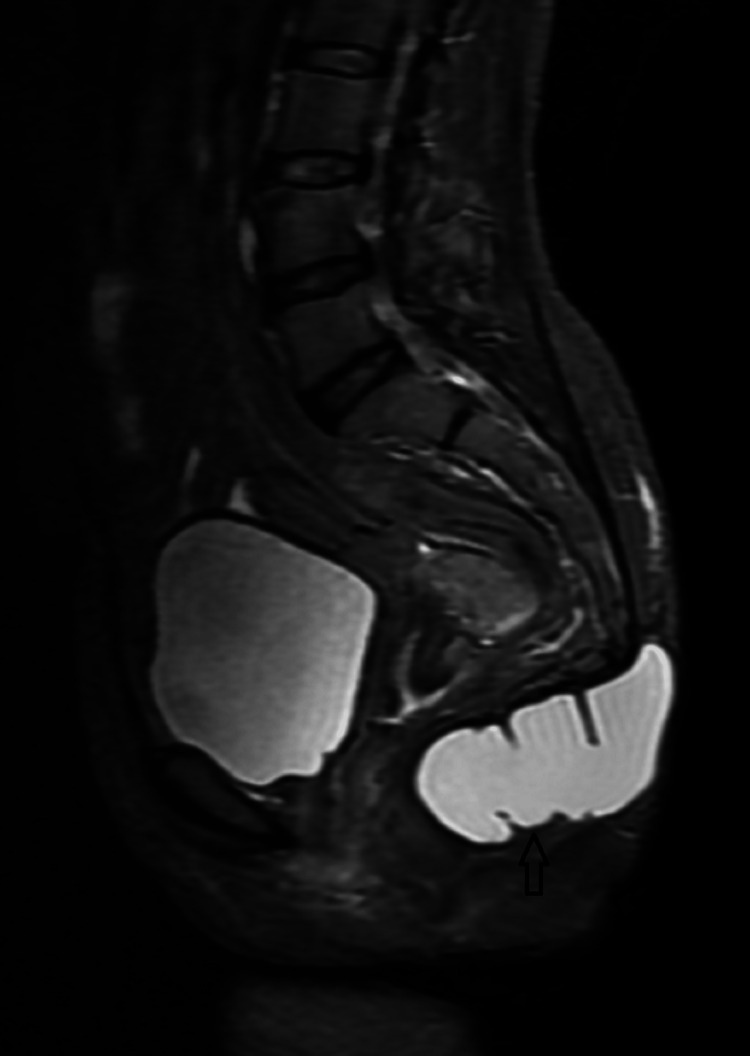
The sagittal section MRI STIR image shows that the lesion is appearing hyperintense (black arrow). STIR: Short tau inversion recovery https://assets.cureus.com/uploads/figure/file/450602/lightbox_27ec4db0313c11eda3a99f5a5f32899d-E3.png

**Figure 5:**
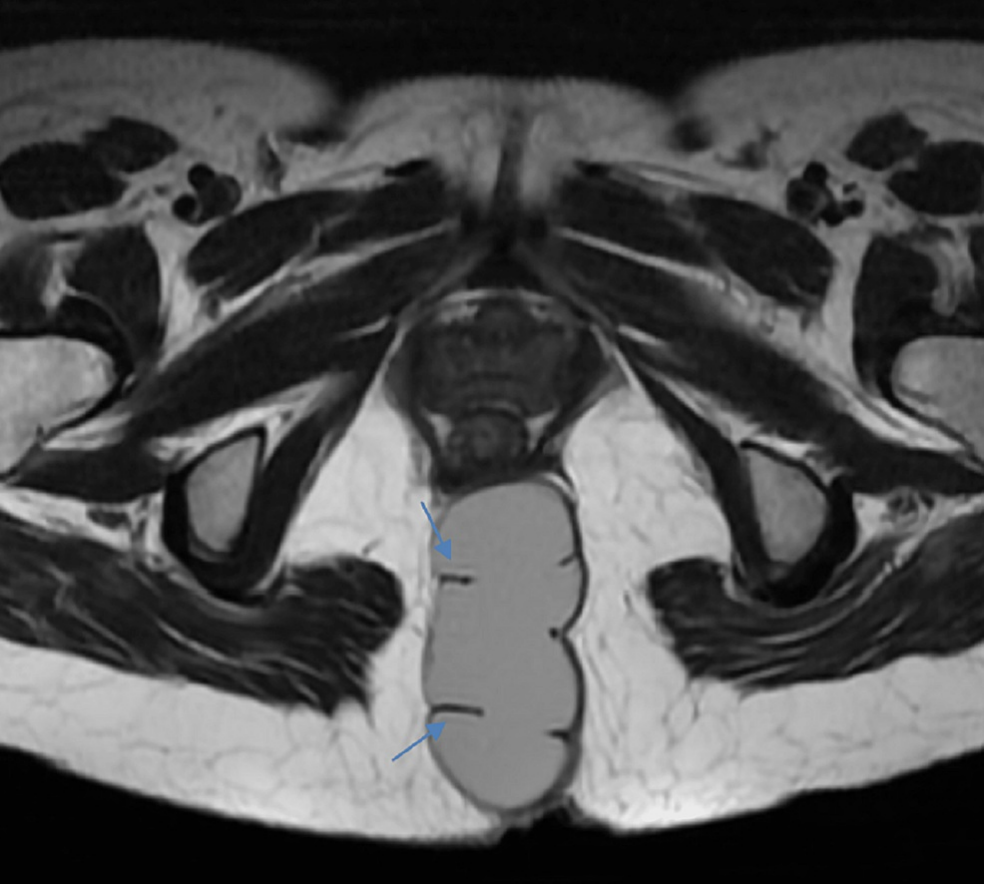
Axial MRI T2WI images showing well-defined hyperintense lesions showing incomplete septations within (blue arrow). T2WI: T2 weighted image https://assets.cureus.com/uploads/figure/file/450603/lightbox_010afed0313c11ed8339570870798b63-E4.png

**Figure 6:**
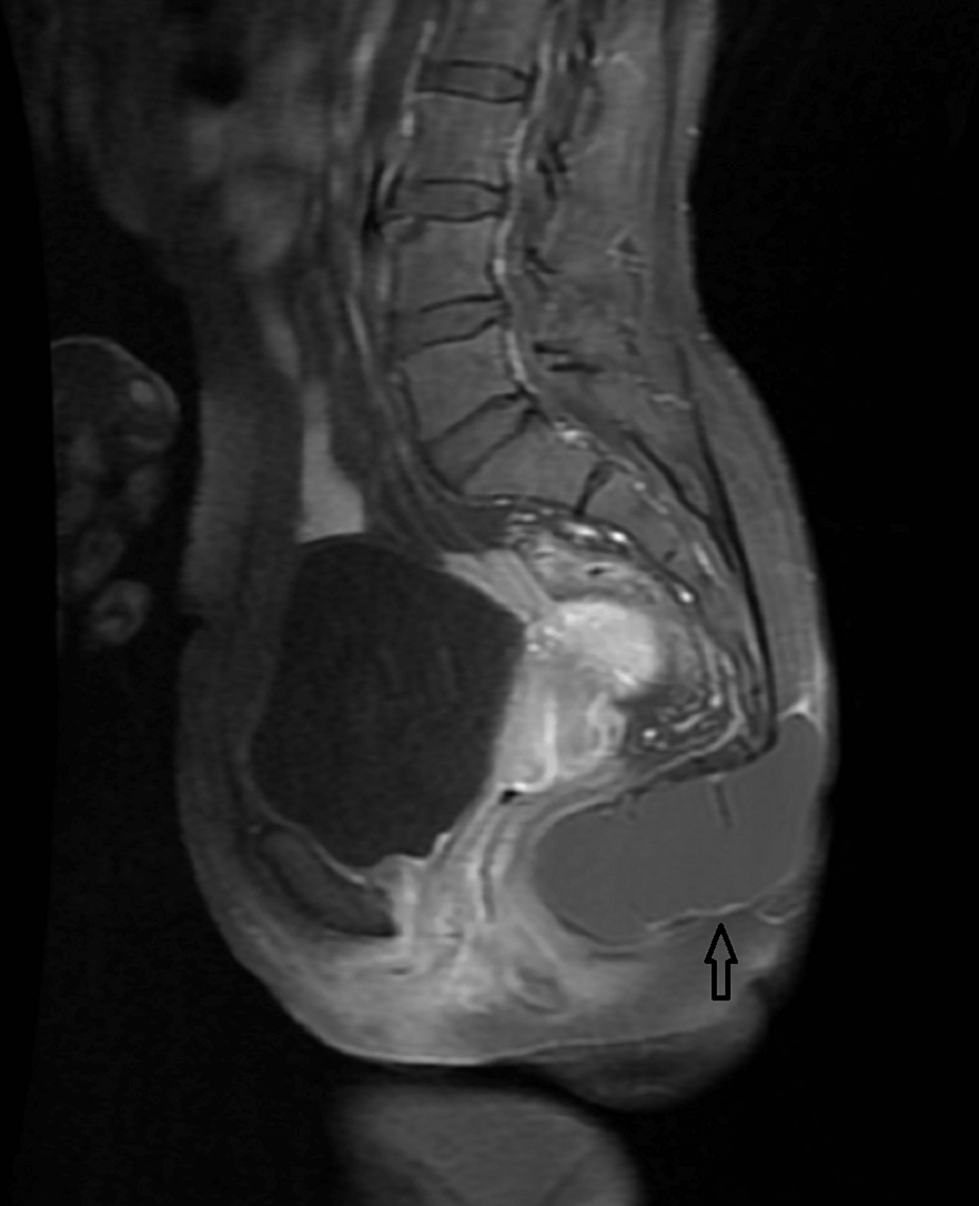
Contrast-enhanced T1WI sagittal MRI image showing subtle peripheral enhancement (black arrow). T1WI: T1 weighted image https://assets.cureus.com/uploads/figure/file/450604/lightbox_d7c86760313b11ed8c9887c19bb9f4af-E5.png

## Discussion

Epidermal cysts are keratin-rich and linked with stratified squamous epithelia. The mitosis of epidermal cells within the closed cavitation of the dermis is involved in the pathophysiology of epidermal cysts. Men are nearly twice as likely as women to have epidermoid cysts. They can occur at any age; although, statistically it is most common in the third to fourth decades of life. Epidermal cysts usually occur on the face, head, neck, and torso; perineal involvement is rare. Only a few cases of unanticipated intra-cystic bleeding and epidermoid cyst malignancy have been described in the literature [6,7].

US is often the initial modality of choice in evaluating masses in the perianal region. Selected soft-tissue lesions can be diagnosed based on certain clinical and imaging features. Epidermoid cysts are rather common on ultrasonography, but they may have a non-specific and hazy appearance, and variable imaging features require additional imaging. Ultrasound is useful for determining the anatomic position and proximity to neighboring structures, and features like echogenicity, size, and margination can be routinely seen [8]. Epidermoid cysts have classic cystic characteristics; however, they can be solid or cystic. They're round to oval, with a well-defined avascular mass in the dermis and subcutaneous tissue, as well as dorsal acoustic amplification and lateral shadowing [9].

Both CT and MRI are utilized to identify epidermoid cysts and define the surgical removal procedure. CT is useful for confirming the diagnosis of a large epidermoid cyst, but not for small epidermoid cysts. On CT, a well-encapsulated mass of heterogeneous densities can be detected, which reflects a mixture of fat and keratin [10].

The MRI findings of epidermal inclusion cysts are influenced by the cyst's maturity, compactness, and the amount of keratin it contains [11]. Epidermoid cysts emerge as T1 hypointense, and T2 hyperintense masses with diffusion restriction when located throughout the body. T2 hypointense foci may occur within the lesion due to the presence of keratin [12]. Most epidermoid cysts do not display contrast enhancement; however, rim enhancement can be found in 25% of instances [13].

## Conclusions

MRI is one of the best investigations of choice to evaluate perineal lesions, which helps in understanding the nature as well as the extent and involvement of the adjacent soft tissue with its fine resolution. Epidermoid cysts that have not ruptured typically show well-defined mass lesions with low signal intensity, some bright foci on T1-weighted MR images, and high signal intensity on T2-weighted MR imaging.
